# Deep-water parasite diversity in Lake Tanganyika: description of two new monogenean species from benthopelagic cichlid fishes

**DOI:** 10.1186/s13071-016-1696-x

**Published:** 2016-08-03

**Authors:** Nikol Kmentová, Milan Gelnar, Stephan Koblmüller, Maarten P. M. Vanhove

**Affiliations:** 1 Department of Botany and Zoology, Faculty of Science, Masaryk University, Kotlářská 2, 611 37 Brno, Czech Republic; 2 Institute of Zoology, University of Graz, Universitätsplatz 2, A-8010 Graz, Austria; 3 Institute of Vertebrate Biology, Academy of Sciences of the Czech Republic, Květná 8, 603 65 Brno, Czech Republic; 4 Biology Department, Royal Museum for Central Africa, Leuvensesteenweg 13, B-3080 Tervuren, Belgium; 5 Laboratory of Biodiversity and Evolutionary Genomics, Department of Biology, University of Leuven, Ch. Deberiotstraat 32, B-3000 Leuven, Belgium; 6 Present address: Capacities for Biodiversity and Sustainable Development, Operational Directorate Natural Environment, Royal Belgian Institute of Natural Sciences, Vautierstraat 29, B-1000 Brussels, Belgium

**Keywords:** *Benthochromis horii*, Cichlidae, *Cichlidogyrus*, Monogenea, *Trematocara unimaculatum*

## Abstract

**Background:**

Lake Tanganyika is the world’s second deepest lake. Its diverse cichlid assemblage offers a unique opportunity for studying a deep-water host-parasite model in freshwater. Low host specificity and a broad host range including representatives of the Bathybatini tribe in the only monogenean parasite described from this habitat, *Cichlidogyrus casuarinus* Pariselle, Muterezi Bukinga & Vanhove, 2015 suggest a link between lower specificity and lower host density. Conversely, high host specificity and species richness are reported for monogeneans of the lake’s littoral cichlids. We further investigated whether the deep-water environment in Lake Tanganyika is really monogenean species-depauperate by investigating the monogenean fauna of *Trematocara unimaculatum* (a representative of the tribe Trematocarini, the sister lineage of the Bathybatini) and *Benthochromis horii*, a member of the tribe Benthochromini, found in the same deep-water habitat as the already known hosts of *C. casuarinus*.

**Methods:**

Sclerotised structures of the collected monogenean individuals were characterised morphologically using light microscopy and morphometrics.

**Results:**

Both examined cichlid species are infected by a single monogenean species each, which are new to science. They are described as *Cichlidogyrus brunnensis* n. sp., infecting *T. unimaculatum*, and *Cichlidogyrus attenboroughi* n. sp., parasitising on *B. horii*. Diagnostic characteristics include the distal bifurcation of the accessory piece in *C. brunnensis* n. sp. and the combination of long auricles and no heel in *C. attenboroughi* n. sp. In addition *C. brunnensis* n. sp. does not resemble *C. casuarinus*, the only species of *Cichlidogyrus* thus far reported from the Bathybatini. Also *Cichlidogyrus attenboroughi* n. sp. does not resemble any of the monogenean species documented from the pelagic zone of the lake and is among the few described species of *Cichlidogyrus* without heel.

**Conclusions:**

As two new and non-resembling *Cichlidogyrus* species are described from *T. unimaculatum* and *B. horii*, colonisation of the deep-water habitat by more than one morphotype of *Cichlidogyrus* is evident. Based on morphological comparisons with previously described monogenean species, parasite transfers with the littoral zone are possible. Therefore, parasites of pelagic cichlids in the lake do not seem to only mirror host phylogeny and the evolutionary history of this host-parasite system merits further attention.

## Background

Considering the high number of vertebrate [1–4] and invertebrate [5–11] radiations described from Lake Tanganyika, it is surprising that its parasite fauna has been mostly overlooked for many years. Parasitological research in Lake Tanganyika has increased since about five years, improving our understanding of mainly its monogenean fauna [12–17]. The Monogenea van Beneden, 1858 is a group of mostly ectoparasitic flatworms described mainly from freshwater fishes and phylogenetically closely related to Cestoda van Beneden, 1849 [18, 19]. Due to their direct life-cycles and high degree of structural adaptations influenced by host preferences, they are considered as useful targets for investigations focusing on evolutionary processes in parasites [20–23] as well as for research on the taxonomy [16], biogeography [24–26] or phylogeny of their host species [27]. This is nicely exemplified when focusing on the cichlid fishes (family Cichlidae), one of the most diverse host fish families with a remarkable evolutionary history featuring rapid radiation processes [1, 28, 29]. Cichlids display huge species richness and are usually classified into tribes [30].

Lake Tanganyika in the African Rift Valley is the second deepest lake in the world and figures as a natural experiment displaying the greatest diversity of speciation mechanisms in cichlids compared to the other major African lakes [28, 31]. Lake Tanganyika is inhabited by more than 200 cichlid species [31], classified into 15 different tribes [32–34]. Six monogenean genera have been reported to infect African cichlid fishes. *Cichlidogyrus* Paperna, 1960 is the most species-rich one [15, 24, 35]. To date, 22 species of *Cichlidogyrus* have been described in Lake Tanganyika from 18 cichlid hosts representing seven different tribes [12–17]. Most records originated from the littoral zone where these parasites were shown to display quite strong host specificity [27]. Cichlid species richness in Lake Tanganyika decreases with water depth [31], with most of the diversity found in shallow littoral habitats. This situation is caused by three main factors: reduction of niche diversity in the deep-water habitat, the fact that the short-wave length blue light spectrum in the depths does not promote diversification mechanisms, and the absence of strong geographic barriers [36–39]. The same pattern of lower species richness, along with lower host specificity, was documented in monogeneans and suggested to be a consequence of lower host availability. For example, the generalist *C. casuarinus* Pariselle, Muterezi Bukinga & Vanhove, 2015 has been reported from six bathybatine cichlid species belonging to the genera *Bathybates* and *Hemibates* [13, Kmentová et al., unpublished observation]. To further our knowledge of the monogenean diversity in deep-water Tanganyika cichlids, we examined *Trematocara unimaculatum* Boulenger, 1901, a representative of the tribe Trematocarini, the sister group of the Bathybatini [34, 40], and *Benthochromis horii* Takahashi, 2008, a cichlid species belonging to another deep-water tribe, Benthochromini, which is only distantly related to the Bathybatini [31, 34], but found in the same habitat as the previously reported hosts of *C. casuarinus*.

## Methods

Fishes were bought on fish markets in the capital city of Burundi, Bujumbura (3°23'S, 29°22'E) in September 2013 and identified in situ. Eight specimens of *Trematocara unimaculatum* and ten of *Benthochromis horii* were dissected according to the standard protocol of Ergens & Lom [41]. Gills were preserved in ethanol until subsequent inspection for monogeneans under an Olympus SYX7 stereomicroscope. Parasites were mounted on slides under coverslips using Hoyer’s medium, enabling visualisation of sclerotised structures [42]. Measurements of sclerotised structures were taken at a magnification of 1000× (objective × 100 immersion, ocular × 10) using an Olympus BX51 microscope with incorporated phase contrast and the software MicroImage version 4.0. In total, 26 different metrical features were measured and are presented in micrometres. The terminology follows [17, 43] while “straight length” and “straight width” mean the linear length of the measured structure. Drawings were made using an Olympus BX51 microscope equipped with a drawing tube and OLYMPUS KL 1500 LED illumination and edited with a graphics tablet compatible with Adobe Illustrator 16.0.0 and Adobe Photoshop 13.0. The type-material was deposited in the invertebrate collection of the Royal Museum for Central Africa (RMCA), Tervuren, Belgium; the Iziko South African Museum (SAMC), Cape Town, Republic of South Africa; the Muséum national d’Histoire naturelle (MNHN), Paris, France; and the Natural History Museum (NHMUK), London, United Kingdom. Tissue samples of the hosts are available in the ichthyology collection of the RMCA.

## Results

*Trematocara unimaculatum* and *B. horii* were each infected by a single different monogenean species belonging to *Cichlidogyrus.* Following Paperna [44] and Pariselle et al. [45], the genus is characterised by a haptor consisting of two pairs of medium-sized anchors, seven pairs of marginal hooks, two transversal bars (the dorsal one with two auricles; the ventral one curved and articulated), a male copulatory organ (MCO) with copulatory tube and most often a heel and (see [15]) an accessory piece; and a vagina which is not always sclerotised. Both collected *Cichlidogyrus* spp. are new to science and their descriptions are presented below. Since the species description in dactylogyrid monogenean taxonomy is more than anything else based on the morphology of their sclerotised structures [19, 46], the depiction of soft parts and internal organs is omitted and a differential diagnosis focused on details of the parasites’ hard parts is provided.

**Family Dactylogyridae Yamaguti, 1963**

**Genus*Cichlidogyrus*Paperna, 1960**

***Cichlidogyrus brunnensis*n. sp.**

***Type-host***: *Trematocara unimaculatum* Boulenger, 1901 (Cichlidae).

***Type-locality*:** Bujumbura, Lake Tanganyika, Burundi (3°23'S, 29°22'E), coll. 4.ix.2013.

***Type-material***: Holotype: MRAC MT.37812. Paratypes: MRAC MT.37812-4 (16 specimens); MNHN HEL549-550 (4 specimens); NHMUK 2015.12.10.1-2 (3 specimens); SAMC-A082649-50 (3 specimens).

***Site in host***: Gills.

***Infection parameters***: Five of eight fish infected with 2–23 specimens.

***ZooBank registration***: To comply with the regulations set out in article 8.5 of the amended 2012 version of the *International Code of Zoological Nomenclature* (ICZN) [47], details of the new species have been submitted to ZooBank. The Life Science Identifier (LSID) of the article is urn:lsid:zoobank.org:pub:F7E8CC4E-8B91-48A9-9131-3BBBC80F798F. The LSID for the new name *Cichlidogyrus brunnensis* is urn:lsid:zoobank.org:act:270003FD-6002-404B-BD38-37F5ED161EEA.

***Etymology***: The species epithet was chosen after the biggest Moravian city, Brno, Czech Republic, where Masaryk University was founded, in gratitude for the education and support provided.

### Description

**Fig. 1:**
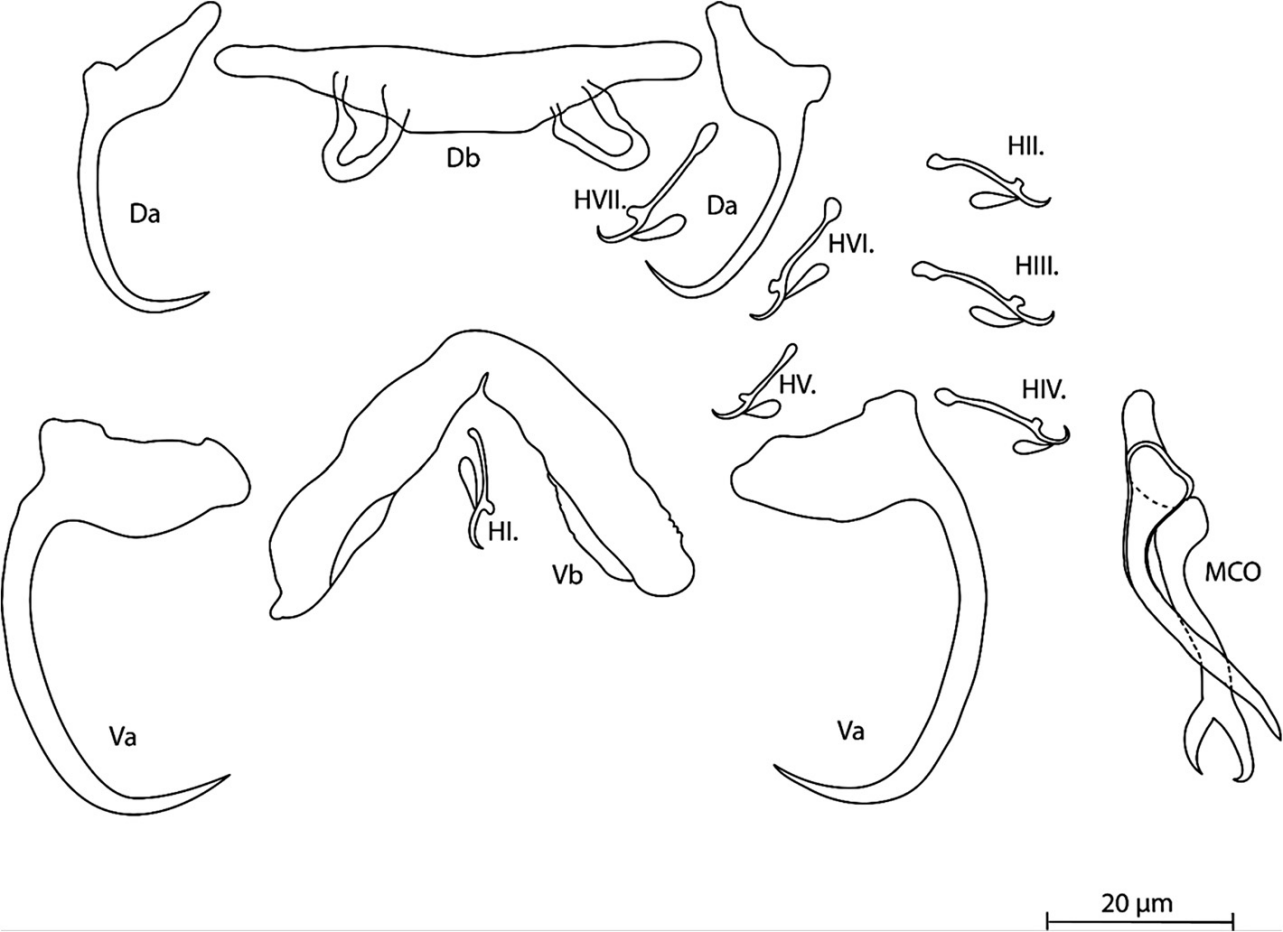
Sclerotised structures of *Cichlidogyrus brunnensis* n. sp. ex *Trematocara unimaculatum*. *Abbreviations*: Da, dorsal anchors; Db, dorsal bar; H, hooks; MCO, male copulatory organ; Va, ventral anchors; Vb, ventral bar

[Based on 30 specimens; Figs. 1, 3a, c; see measurements in Table 1.] Dorsal anchor with poorly incised roots and well-developed, regularly curved short point. Ventral anchors larger than dorsal anchors, with longer distance between base and point of hook, poorly incised roots, short point. Dorsal bar large, wide, straight, with relatively short, wide auricles. Ventral bar thick, short, branches straight with constant width. Hooks 7 pairs, pairs 1–4, 6 and 7 relatively short (*sensu* [35]) compared to pair 5, considering ontogenetic development as pair 5 retains its larval size); pair 7 largest. MCO small, with narrow, thin-walled tubular copulatory tube; accessory piece of same length as copulatory tube with distal bifurcation starting in distal quarter, and short heel. Sclerotised vagina not observed.

**Table 1:** Measurements (in micrometres) for the two new species of *Cichlidogyrus* described in this study

Character	*C. brunnensis* n. sp. (*n* = 30)			*C. attenboroughi* n. sp. (*n* = 30)		
	Range	Mean	*n*	Range	Mean	*n*
Total body length	493.9–772.5	657.1	3	436–344.7	378.9	10
Ventral anchor						
Total length	38.1–42.7	41.6	25	30.2–34.5	32.8	21
Length to notch	36.6–45.9	40.0	24	25.2–30.4	27.4	18
Inner root length	3.3–15.2	11.8	19	5.9–10.4	8.0	18
Outer root length	2.4–7.6	5.4	11		4.6	12
Point length	10.6–16.0	13.5	23	3.3–5.3	8.2	
Dorsal anchor						
Total length	30.2–36.6	33.1	20	25.8–33.6	29.9	23
Length to notch	25.0–29.4	27.1	19	18.3–26.5	23.0	20
Inner root length	5.6–12.7	10.5	19	7.3–11.2	9.2	21
Outer root length	0.9–5.0	4.0	16	3.4–8.0	5.1	18
Point length	7.3–10.0	8.7	20	6.0–8.8	7.4	17
Ventral bar						
Branch length	32.3–48.2	38.5	19	30.4–41.0	34.7	26
Branch maximum width	8.2–10.4	9.2	22	4.0–5.8	5.1	28
Dorsal bar						
Maximum straight width	35.8–51.1	43.5	15	31.8–44.6	38.1	24
Thickness at midlength	6.8–10.6	8.2	23	4.1–6.7	5.6	28
Distance between auricles	14.6–22.2	17.4	21	9.3–15.5	13.0	23
Auricle length	5.7–14.8	11.1	15	13.6–23.1	18.4	14
Hooks						
First pair	13.7–19.2	16.2	11	11.1–16.8	11.9	28
Second pair	14.8–18.7	16.1	14	12.3–18.1	16.3	25
Third pair	15.5–20.9	17.6	18	13.4–21.9	17.9	22
Fourth pair	14.1–18.4	16.5	12	19.9–23.4	21.5	15
Fifth pair	11.2–15.4	13.3	11	10.2–14.1	11.6	11
Sixth pair	15.1–27.6	18.0	9	18.4–22.9	20.3	11
Seventh pair	17.0–23.0	18.7	9	15.0–22.5	17.2	10
MCO straight length	40.1–69.4	48.6	17		–	
Copulatory tube curved length	24.5–45.3	37.8	16	47.4–64.5	52.8	27
Accessory piece curved length	29.6–44.8	37.2	14	35.9–53.2	43.4	22
Heel straight length	10.0–4.8	7.6	28		–	

### Differential diagnosis

The anchors of this species resemble those of its non-Tanganyika congeners *Cichlidogyrus sclerosus* Paperna & Thurston, 1969 [48], *C. amphoratus* Pariselle & Euzet, 1996 [49] and *C. giostrai* Pariselle, Bilong Bilong & Euzet, 2003 [45] described from *Oreochromis mossambicus* (Peters, 1852), *Tilapia louka* Thys van den Audenaerde, 1969 and *Sarotherodon caudomarginatus* (Boulenger, 1916), respectively, in their broad base and almost non-incised roots of the anchors. However, the exceptional shape of its accessory piece, with a forked ending, as well as the large ventral anchor in comparison to the dorsal one, make *C. brunnensis* n. sp. clearly distinguishable among all species of *Cichlidogyrus* described so far. Moreover, the shape of the anchors, specifically their poorly incised roots and the proportionally large hook, is unique among all other known congeners in Lake Tanganyika: *Cichlidogyrus vandekerkhovei* Vanhove, Volckaert & Pariselle, 2011; *C. makasai* Vanhove, Volckaert & Pariselle, 2011; *C. sturmbaueri* Vanhove, Volckaert & Pariselle, 2011; *C. centesimus* Vanhove, Volckaert & Pariselle, 2011; *C. gillardinae* Muterezi Bukinga, Vanhove, Van Steenberge & Pariselle, 2012; *C. mbirizei* Muterezi Bukinga, Vanhove, Van Steenberge & Pariselle, 2012; *C. nshomboi* Muterezi Bukinga, Vanhove, Van Steenberge & Pariselle, 2012; *C. mulimbwai* Muterezi Bukinga, Vanhove, Van Steenberge & Pariselle, 2012; *C. muzumanii* Muterezi Bukinga, Vanhove, Van Steenberge & Pariselle, 2012; *C. steenbergei* Gillardin, Vanhove, Pariselle, Huyse & Volckaert, 2012; *C. gistelincki* Gillardin, Vanhove, Pariselle, Huyse & Volckaert, 2012; *C. irenae* Gillardin, Vanhove, Pariselle, Huyse & Volckaert, 2012; *C. buescheri* Pariselle & Vanhove, 2015; *C. schreyenbrichardorum* Pariselle & Vanhove, 2015; *C. vealli* Pariselle & Vanhove, 2015; *C. banyankimbonai* Pariselle & Vanhove, 2015; *C. muterezii* Pariselle & Vanhove, 2015; *C. raeymaekersi* Pariselle & Vanhove, 2015; *C. georgesmertensi* Pariselle & Vanhove, 2015; *C. franswittei* Pariselle & Vanhove, 2015; *C. frankwillemsi* Pariselle & Vanhove, 2015 and *C. casuarinus*. The latter species infects bathybatine cichlids, the sister group of the Trematocarini, which among others also comprises *T. unimaculatum*, the type-host of *C. brunnensis* n. sp., but *C. casuarinus* is morphologically very different; it is easily distinguished by the spirally-coiled thickening in the wall of the copulatory tube. Although the forked shape of the accessory piece of *C. brunnensis* n. sp. is similar to an undescribed species collected from *Limnochromis auritus* [50], a member of Lake Tanganyika’s benthic deep water cichlid tribe Limnochomini, differences in haptoral sclerotised structures, namely the length of the dorsal bar auricles (on average 11.1 μm in *C. brunnensis vs c.*45 μm in the undescribed species) and the shape of the anchors allows clear distinction between them.

***Cichlidogyrus attenboroughi*n. sp.**

***Type-host***: *Benthochromis horii* Takahashi, 2008 (Cichlidae).

***Type-locality***: Bujumbura, Lake Tanganyika, Burundi (3°23'S, 29°22'E).

***Type-material***: Holotype: MRAC MT.37815. Paratypes: MRAC: MT.37815-7 (10 specimens); MNHN HEL551-552 (5 specimens); NHMUK 2015.12.10. 3–4 (5 specimens); SAMC-A082651-2 (4 specimens).

***Site in host***: Gills.

***Infection parameters***: Three of ten fish infected with 4–27 specimens.

***ZooBank registration***: To comply with the regulations set out in article 8.5 of the amended 2012 version of the *International Code of Zoological Nomenclature* (ICZN) [47], details of the new species have been submitted to ZooBank. The Life Science Identifier (LSID) of the article is urn:lsid:zoobank.org:pub:F7E8CC4E-8B91-48A9-9131-3BBBC80F798F. The LSID for the new name *Cichlidogyrus attenboroughi* is urn:lsid:zoobank.org:act:AC051EA5-FCAC-49A8-9048-02E44E80654D.

***Etymology***: The species epithet honours the English scientist and broadcaster Sir David Frederick Attenborough, in gratitude for the insights and inspiration he gave to so many people to study and protect nature and biodiversity.

### Description

**Fig. 2:**
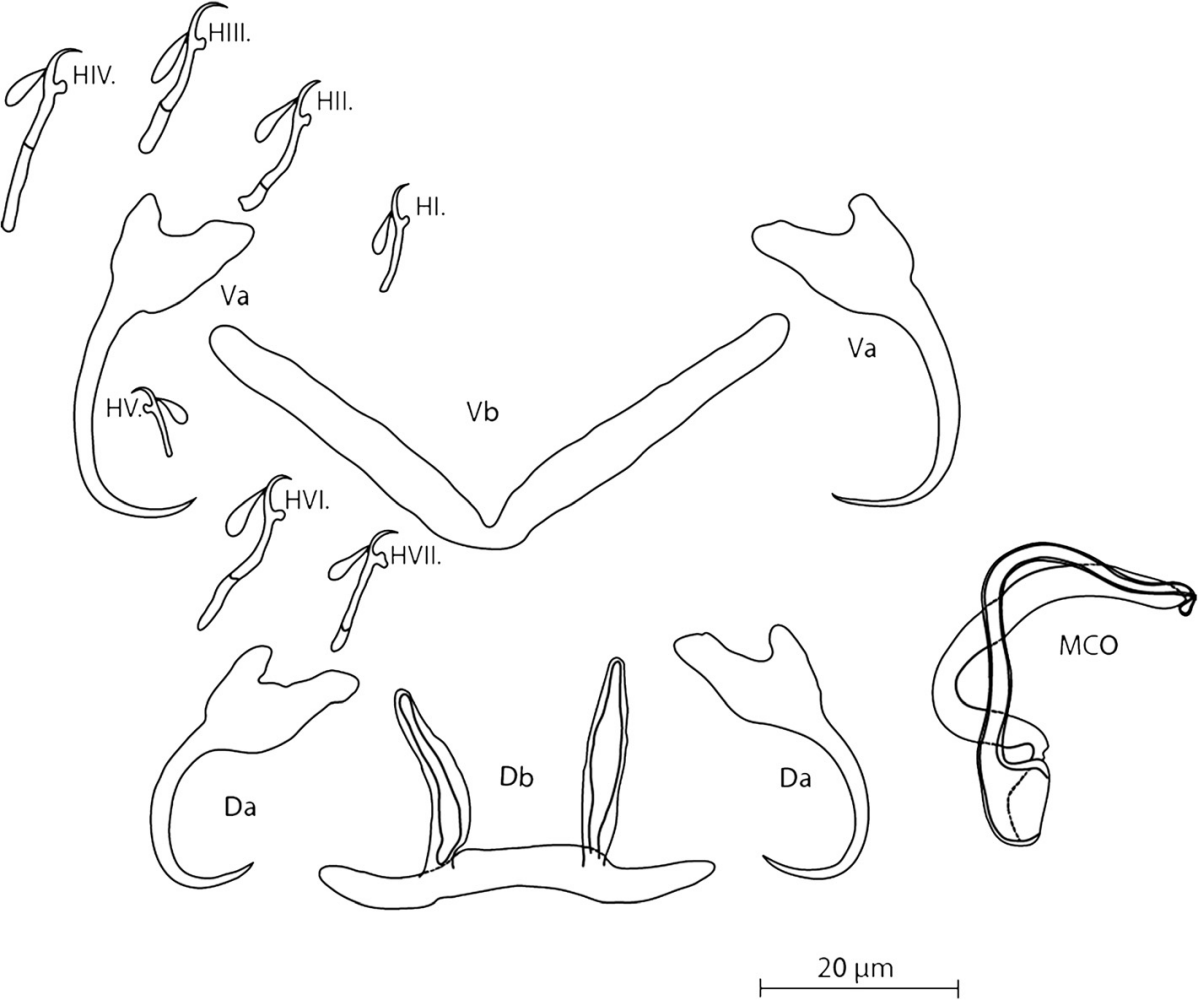
Sclerotised structures of *Cichlidogyrus attenboroughi* n. sp. ex *Benthochromis horii. Abbreviations*: Da, dorsal anchors; Db, dorsal bar; H, hooks; MCO, male copulatory organ; Va, ventral anchors; Vb, ventral bar

**Fig. 3:**
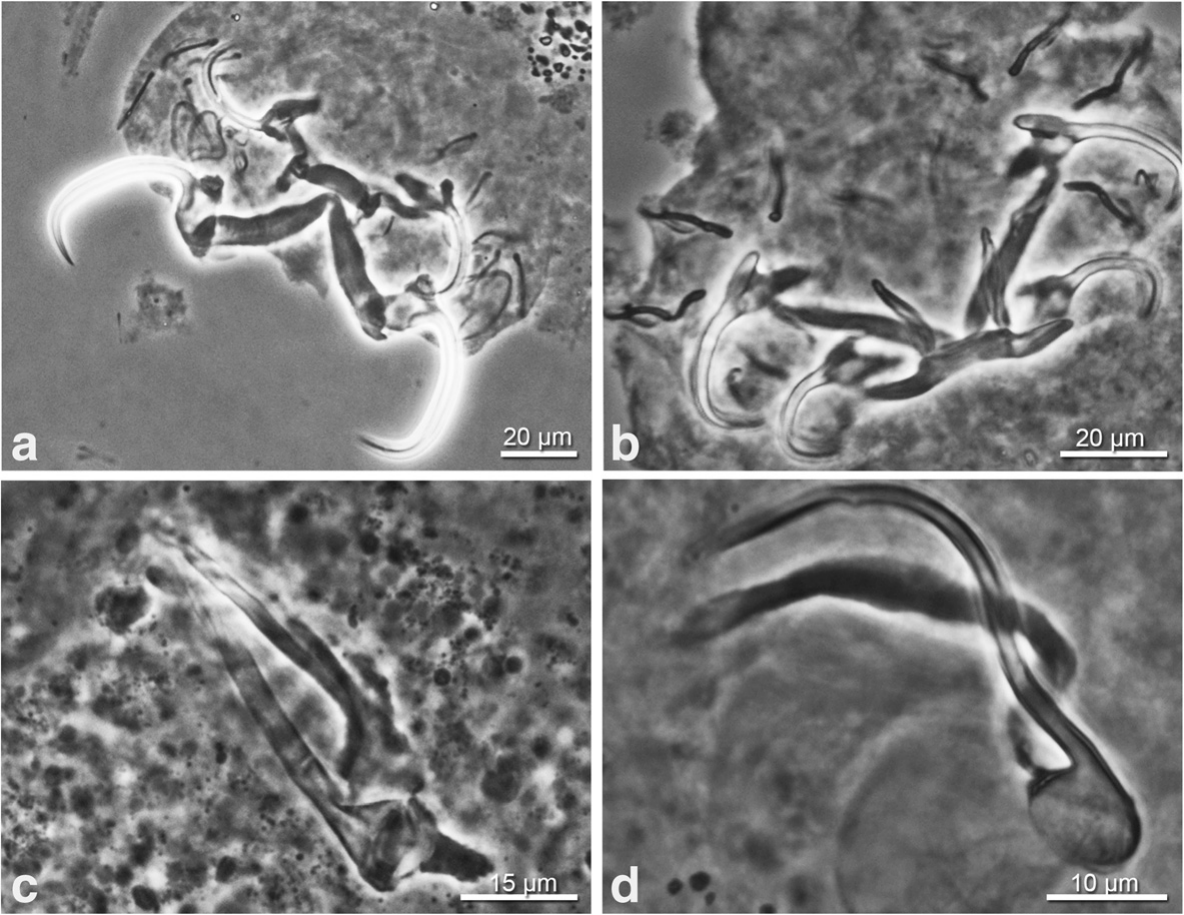
Haptoral and male genital sclerotised structures of *Cichlidogyrus* species described in this study (Hoyer’s medium, phase-contrast photomicrographs). **a** Opisthaptor of *C. brunnensis* n. sp. **b** Opisthaptor of *C. attenboroughi* n. sp. **c** Male copulatory organ of *C. brunnensis* n. sp. **d** Male copulatory organ of *C. attenboroughi* n. sp

[Based on 30 specimens; Figs. 2, 3b, d; see measurements in Table 1.] Dorsal anchors with different root size and regularly curved points. Ventral anchors larger in total size and more similar root size than dorsal anchors. Dorsal bar thin, with relatively long narrow auricles. Ventral bar thin, long, with constant width. Hooks 7 pairs, pair 4 relatively long; pairs 1 and 5 of equal length. MCO with slender copulatory tube with relatively thick wall; accessory piece broader than copulatory tube. No heel or sclerotised vagina observed.

### Differential diagnosis

There are congeners in Lake Tanganyika that share with *C. attenboroughi* n. sp. the small size of the first pair of hooks and the presence of long dorsal bar auricles, namely *C. makasai* and *C. vandekerkhovei* recorded from *Ophthalmotilapia ventralis* (Boulenger, 1898),*O. nasuta* (Poll & Matthes, 1962) and *O. boops* (Boulenger, 1901). *Cichlidogyrus attenboroughi* n. sp. differs from these species by the shorter length of the auricles (18.4 μm in *C. attenboroughi vs* 20 μm in *C. makasai* and 30 μm in *C. vandekerkhovei*) and in possessing a MCO with a simple accessory piece and without a heel. Because of the shape of the ventral anchor and the equal size among the first and the second pairs of hooks this species could be mistaken with *C. gistelincki* infecting *Ctenochromis horei* (Günther, 1894), *C. irenae* described from *Gnathochromis pfefferi* (Boulenger, 1898) and *C. steenbergei* parasitising *Limnotilapia dardennii* (Boulenger, 1899) all of which are also present in Lake Tanganyika. However, in contrast to these species, there is no developed heel in the MCO of *C. attenboroughi* n. sp. Three other species have been described lacking a heel: *C. haplochromii* Paperna & Thurston, [48] described from *Pharyngochromis darlingi* (Boulenger, 1911); *C. tilapiae* Paperna, 1960 recorded from, among others, *Oreochromis leucostictu*s (Trewavas, 1933) and *Sarotherodon galilaeus* (Linnaeus, 1758) and *C. arfii* Pariselle & Euzet, 1995 described from *Pelmatochromis buettikoferi* (Steindachner, 1894). However, the haptoral region of the latter species cannot be confused with *C. attenboroughi* n. sp. because of the different edge of the dorsal anchor roots, the relative length of the first pairs of hooks (the smallest pair in *C. attenboroughi* n. sp. and the biggest pair in *C. arfii*) or the length of the auricles (18.4 μm in *C. attenboroughi* n. sp. *vs* 9 μm in *C. arfii*) [51]. The most evident difference between *C. attenboroughi* n. sp. and *C. tilapiae* is the size of the dorsal anchor as well as the maximal straight width of the dorsal bar and the length of its auricles [45]. Differences with *C. haplochromii* are visible in the dorsal bar, which has shorter and less slender auricles in comparison to *C. attenboroughi* n. sp. [48].

## Discussion

The presence of a single monogenean species, phenotypically substantially different from *C. casuarinus*, on *T. unimaculatum*, indicates that closely related deep-water cichlid lineages have been colonised by several *Cichlidogyrus* lineages. Moreover, comparison with other species reported from Lake Tanganyika so far points to multiple origins of the deep-water representatives of *Cichlidogyrus*. Indeed, this is suggested by the phenotypic similarity of *C. brunnensis* n. sp. to an undescribed species collected from the benthic cichlid *Limnochromis auritus* (Boulenger, 1901) [50] and its quite different morphology of sclerotised structures as compared to *C. attenboroughi* n. sp., which infects another deep-water cichlid species, *Benthochromis horii*. Furthermore, *C. attenboroughi* n. sp. shares morphological characteristics of its haptoral region with two species of *Cichlidogyrus* (*C. vandekerkhovei* and *C. makasai*) recorded from three species of *Ophthalmotilapia* as well as with species described from tropheine cichlids [14, 15]. Interestingly, *C. casuarinus* infecting Bathybatini and *C. nshomboi* infecting *Boulengerochromis microlepis* (Boulenger, 1899), a cichlid species phylogenetically closely related to the Trematocarini and Bathybatini [52–54], are similar to *C. centesimus*. The latter infects three species of the genus *Ophthalmotilapia*, which belongs to the tribe Ectodini. *Cichlidogyrus casuarinus*, *C. nshomboi* and *C. centesimus* share the unique spirally coiled thickening of the wall of the copulatory tube [12, 13, 15]. Both ectodine and tropheine cichlids occur in shallow water and are only distantly related to Bathybatini*,* Trematocarini, Benthochromini and *Boulengerochromis*. Therefore, other scenarios such as host habitat preferences influencing the chance of transmission [55] or shared morphological characters of the host affecting monogenean phenotypes might have played a role in the evolutionary history of this monogenean assemblage [56, 57].

## Conclusions

The inventory of monogeneans from Lake Tanganyika has been supplemented by the description of *C. brunnensis* n. sp. and *C. attenboroughi* n. sp. collected from *T. unimaculatum* and *B. horii*, respectively. These are the first monogeneans reported from the respective cichlid species and tribes. The known host range of *C. casuarinus* still remains limited to the genera *Bathybates* and *Hemibates*, although further investigations are needed to confirm this observation. Our result is consistent with taxonomic hypotheses that include the Trematocarini as a separate tribe [34, 58], which, together with the Bathybatini, constitute the sister group of the Boulengerochromini [53]. *Cichlidogyrus casuarinus*, parasitising bathybatine cichlids, morphologically resembles *C. nshomboi* (collected from *B. microlepis*, see [12, 13]) more than *C. brunnensis* n. sp. which infects *T. unimaculatum*, a representative of the Bathybatini’s sister group Trematocarini. Hence, probably other speciation mechanisms rather than co-speciation have occurred in the evolutionary history of this deep-water parasite-host system. An exhaustive list of *Cichlidogyrus* species occurring on deep-water cichlid species in Lake Tanganyika, together with genetic analyses and a co-phylogenetic approach, are needed to verify these alternative scenarios. The reported lower monogenean host specificity is probably correlated with small diversity and population densities of hosts [58–60] influenced by lower temperature and reduction of light as communication of cichlids is mainly based on visual signals [61]. Decline of parasite diversity is probably also related to the distance from the shore as well as to specific host behavioural characteristics [60]. Although the deep-water monogenean fauna in Lake Tanganyika seems to be less species-rich than the littoral one [27] it is premature to exactly quantify differences in monogenean species richness per host, or to conclude whether the deep-water monogenean fauna is indeed depauperate.

## Abbreviations

MCO, male copulatory organ
